# USEFULNESS OF THE CARDIOPULMONARY EXERCISE TEST UP TO THE ANAEROBIC THRESHOLD FOR PATIENTS AGED ≥ 80 YEARS WITH CARDIOVASCULAR DISEASE ON CARDIAC REHABILITATION

**DOI:** 10.2340/jrm.v56.19453

**Published:** 2024-06-19

**Authors:** Yuiko YANO, Yasunori SUEMATSU, Takuro MATSUDA, Kai TSUKAHARA, Miki SHIROSAKI, Sakiko MATSUO, Kanta FUJIMI, Shinichiro MIURA

**Affiliations:** 1 Department of Cardiology, Fukuoka University Hospital, Fukuoka; 2 Miyase Clinic, Fukuoka, (present affiliation); 3 Department of Rehabilitation, Fukuoka University Hospital, Fukuoka; 4 Hinoki Clinic, Fukuoka, (present affiliation); 5 Department of Nutrition, Fukuoka University Hospital, Fukuoka; 6 Department of Cardiology, School of Medicine, Fukuoka University, Fukuoka, Japan

**Keywords:** cardiopulmonary exercise test, older patients, exercise guidance

## Abstract

**Objective:**

A cardiopulmonary exercise test provides information regarding appropriate exercise intensity, but there have been few reports on its use in patients over 80 years of age.

**Design:**

Retrospective observational study.

**Patients:**

A total of 511 cardiovascular disease patients who performed a cardiopulmonary exercise test from February 2011 to January 2020 were investigated.

**Methods:**

Patients were stratified according to age: < 70 years, 70–79 years, and ≥ 80 years, and the results of the cardiopulmonary exercise test up to anaerobic threshold were compared.

**Results:**

Patients in the < 70 age bracket showed higher oxygen consumption, carbon dioxide output, and ventilatory volume and lower ventilation equivalents per oxygen consumption and carbon dioxide output in all time periods. However, there were no significant differences in these parameters or the work rate (70–79 years of age: 41.4 ± 11.7 watts, vs ≥ 80 years: 42.2 ± 10.9 watts, *p* = 0.95) or oxygen consumption per body weight at anaerobic threshold (12.2 ± 0.2 ml/min/kg, vs 12.1 ± 0.4 ml/min/kg, *p* = 0.97) between the 70–79 year age bracket and the ≥ 80 year age bracket.

**Conclusion:**

Even for cardiovascular disease patients age ≥ 80 years, a cardiopulmonary exercise test up to anaerobic threshold can supply useful information for guiding cardiac rehabilitation.

LAY ABSTRACT

Basically, a cardiopulmonary exercise test is used to test maximal exercise capacity. However, some older patients with cardiovascular disease cannot achieve their peak exercise capacity and there have been few reports concerning cardiopulmonary exercise tests in patients over the age of 80 years. However, a cardiopulmonary exercise test up to the anaerobic threshold, rather than up to peak oxygen consumption, is still clinically useful for exercise prescription. We stratified 511 cardiovascular disease patients who performed a cardiopulmonary exercise test from February 2011 to January 2020 according to age: < 70 years, 70–79 years, and ≥ 80 years, and compared the results of the cardiopulmonary exercise test under the anaerobic threshold in the 3 age groups. While the heart rate at rest and at warm-up and ventilation equivalents per oxygen consumption and carbon dioxide output at rest decreased with age, there were no significant differences in most of the other parameters, including the work rate and oxygen consumption per bodyweight between the 70–79 year age bracket and the > = 80 year age bracket. Even for cardiovascular disease patients age ≥ 80 years, cardiopulmonary exercise test up to the anaerobic threshold can supply useful information for cardiac rehabilitation, just as it does in patients aged 70–79 years.

The increasing number of patients with heart failure is an international health issue and the concept of a “heart failure pandemic” was proposed in 2010 (1). It is expected that heart failure will become more serious as the population ages (2). In this era, the prevention of frailty and sarcopenia and the maintenance of quality of life are important issues, in addition to treatment of the underlying disease (3–6).

The cardiopulmonary exercise test (CPET) is an incremental exercise load test that is performed in combination with a respiratory gas analysis. Since CPET can measure oxygen consumption, carbon dioxide output and ventilation volume during exercise in real time, it can be used to evaluate exercise tolerance, cardiopulmonary function, peripheral circulation, pulmonary circulation, and skeletomuscular function (7–9). CPET determines an appropriate exercise intensity for training in cardiac rehabilitation and for lifestyle-related disease management (10). While some older adults are unable to tolerate exercise testing at higher intensities, performing CPET to the anaerobic threshold (AT) can still provide useful information for exercise prescription.

As cardiac rehabilitation becomes more common, the demand for CPET in older patients with cardiovascular disease (CVD) is increasing in Japan. However, there are currently few reports on this subject in patients over 80 years of age. In this study, we investigated the results for (a) heart rate (HR), (b) systolic and diastolic blood pressure, (c) oxygen uptake (VO_2_) and carbon dioxide output (VCO_2_), (d) respiratory exchange ratio (RER), (e) metabolic equivalents (METS), (f) VO_2_/ body weight (VO_2_/wt), (g) VO_2_/HR, (h) work rate, (i) ventilator equivalents (VE), (j) tidal volume, (k) respiratory rate (RR), (l) end-tidal oxygen (ETO_2_), (m) end-tidal carbon dioxide (ETCO_2_), (n) VE/VO_2_, (o) VE/VCO_2_, (p) dead-space gas volume to tidal volume ratio (DV/DT) at rest, warmup, 1 min before AT, and at AT in patients with CVD, including those over 80 years of age.

## METHODS

### Study population and protocol

This single-centre retrospective study evaluated the results of CPET up to AT by age group in patients with CVD. Between February 2011 and January 2020, 511 patients aged 16–88 years performed CPET at Fukuoka University Hospital; when a patient performed CPET multiple times, we selected the most recent result. We stratified the patients into 3 groups according to age: under 70 years (*n* = 303), 70–79 years (*n* = 149), and ≥ 80 years (*n* = 59) and compared the results of CPET under AT between the groups. This study was approved by the ethics committee of Fukuoka University Hospital (U20-10-004). The study was performed in accordance with the Declaration of Helsinki and the ethical standards of the Independent Review Board of Fukuoka University.

### CPET

CPEX1 (Inter Riha, Tokyo, Japan) was used as an expired gas analyser and a Strength Ergo 8 cycle ergometer (Fukuda Denshi, Tokyo, Japan) was used as a stress test system. CPET was performed according to the following protocol: a 4-min rest period was followed by a 4-min warmup and a ramp protocol period. Most patients performed a ramp protocol with an increase of 10 watts per minute. A symptom-limited exercise test was then performed. We analysed HR, systolic blood pressure, diastolic blood pressure, VO_2_, VCO_2_, RER, METS, VO_2_/wt, VO_2_/HR, work rate (watts), VE, tidal volume, RR, ETO_2_, ETCO_2_, VE/VO_2_, VE/VCO_2_, and DV/DT at rest, during warmup, 1 min before AT, and at AT. AT was considered to occur when a disproportionate increase in VCO_2_ against VO_2_ occurred (V-slope method) (11). We did not analyse the parameters over the AT period or the results from interval analysis using the peak period, such as the VE/VCO_2_ slope, because many older patients showed a lower respiratory exchange ratio.

### Data collection

Age, sex, body mass index, and history or presence of smoking were recorded. Comorbidities included hypertension, diabetes mellitus, dyslipidaemia, chronic kidney disease, chronic obstructive pulmonary disease, and other pulmonary disease. CVD included ischaemic heart disease, chronic heart failure, and major vascular disease. Medications included calcium channel blocker, renin angiotensin aldosterone system, diuretics, beta blocker, and mineral corticoid receptor antagonist. Treatments included percutaneous coronary intervention, coronary artery bypass grafting, implantation of an implantable cardioverter defibrillator or cardiac resynchronization therapy, and cardiac operation. The results of echocardiography and the plasma level of brain natriuretic peptide (BNP) were obtained if the examinations were performed within 3 months from the day of CPET. BNP was measured by the chemiluminescent enzyme immunoassay method at Fukuoka University Hospital. Echocardiography was performed using Vivid E 95 (GE HealthCare Japan, Tokyo, Japan), ARIETTA 850 (Fujifilm, Tokyo, Japan), EPIQ 7G (Philips Japan, Tokyo, Japan), and Aplio 400 (Canon Medical Systems Corporation, Tochigi, Japan) at Fukuoka University Hospital. The results of echocardiography were used to determine the left atrium diameter, left ventricular end-diastolic diameter (LVDd), left ventricular end-systolic diameter (LVDs), left ventricular ejection fraction (LVEF), stroke volume, and tricuspid regurgitation peak gradient (TRPG).

### Statistical analysis

All of the data analyses were performed using the SAS (Statistical Analysis System) Software Package (Ver. 9.4, SAS Institute Inc, Cary, NC, USA) at Fukuoka University (Fukuoka, Japan). Continuous variables with a non-normal distribution are presented as the median and interquartile range. Categorical variables are presented as number (%). We investigated the differences between the three age groups by one-way analysis of variance for continuous variables and by the Kruskal–Wallis test for categorical variables with a post-hoc comparison. A value of *p* < 0.05 was considered significant.

## RESULTS

### Characteristics of patients in the 3 age groups

The patient characteristics are listed in Table I. Overall, the median patient age was 67 (56–74) years, 70.5% were males, and the body mass index was 23.5 (21.5–26.2) kg/m^2^. The percentages of males and body mass index in the 70–79 year age group were lower than those in the < 70 group. There were no significant differences between the 70–79 year age group and ≥ 80 years group. With regard to the underlying disease and treatment, the percentages of patients with chronic heart failure, percutaneous coronary intervention, and cardiac operation in the ≥ 80 years group were higher, higher, and lower than those in the 70–79 year age group, respectively.

**Table I:** Baseline characteristics in the 3 age groups

Variables	< 70 years		70–79 years		80 years and above	
	Missing number	(*n* = 303)	Missing number	(*n* = 149)	Missing number	(*n* = 59)
Age, years	0	58 (49–65)	0	74 (72–76)*	0	82 (81–85)*†
Male, *n* (%)	0	224 (73.9)	0	94 (63.1)*	0	42 (71.2)
Body mass index, kg/m^2^	0	23.8 (21.6–26.6)	0	23.1 (21.3–25.5)*	0	23.1 (21.4–25.6)
Smoking, *n* (%)	33	204 (75.6)	14	90 (66.7)	4	28 (50.9)*
Hypertension, *n* (%)	1	165 (54.6)	1	98 (66.2)	0	44 (74.6)
Diabetes mellitus, *n* (%)	1	93 (30.8)	1	49 (33.1)	0	14 (23.7)
Dyslipidaemia, *n* (%)	1	175 (57.9)	1	99 (66.9)	0	40 (67.8)
Chronic kidney disease, *n* (%)	1	81 (26.8)	1	80 (54.1)*	0	38 (64.4)*
COPD, *n* (%)	1	9 (3.0)	1	9 (6.1)*	0	2 (3.4)
Other pulmonary disease, *n* (%)	1	34 (11.3)	1	30 (20.3)	0	10 (16.9)
Ischaemic heart disease, *n* (%)	1	120 (39.7)	1	75 (50.3)	0	36 (61.0)*
Chronic heart failure, *n* (%)	1	118 (39.1)	1	57 (38.5)	0	36 (61.0)*†
Major vessel disease, *n* (%)	1	14 (4.6)	1	17 (11.5)	0	2 (3.4)
Calcium channel blocker, *n* (%)	22	73 (26.0)	5	67 (46.5)*	3	28 (50.0)*
RAAS, *n* (%)	22	166 (59.1)	5	89 (61.8)	3	42 (75.0)
Diuretics, *n* (%)	22	91 (32.4)	5	39 (27.1)	3	18 (32.1)
Beta blocker, *n* (%)	22	172 (61.2)	5	70 (48.6)*	3	34 (60.7)
MRA, *n* (%)	22	77 (27.4)	5	25 (17.4)	3	10 (17.9)
PCI, *n* (%)	1	95 (31.5)	1	47 (31.8)	0	30 (50.8)*†
CABG, *n* (%)	1	27 (8.9)	1	17 (11.5)	0	4 (6.8)
ICD/CRT, *n* (%)	1	21 (7.0)	1	9 (6.1)	0	6 (10.2)
Cardiac operation, *n* (%)	1	36 (11.9)	1	18 (12.2)	0	1 (1.7)*†

Baseline characteristics in the 3 age groups are shown.

* Indicates a significant difference compared with the < 70 years group.

† Indicates a significant difference compared with the 70–79 years group. COPD: chronic obstructive pulmonary disease; RAAS: renin angiotensin aldosterone system; MRA: mineral corticoid receptor antagonist; PCI: percutaneous coronary intervention; CABG: coronary artery bypass grafting; ICD: implantable cardiac defibrillator; CRT: cardiac resynchronization therapy.

### Results of cardiac examinations by echocardiography and plasma examinations

The results of cardiac examinations are given in Table II. There were some missing variables, due to the retrospective nature of this study. Overall, LVEF was 59.6 (46.1–67.1)%, LVDd was 48.0 (43.5–54.7) mm, and TRPG was 22.0 (17.0–26.8) mmHg by echocardiography. The level of plasma BNP was 69.1 (27.7–200.1) pg/ml. LVDd and LVDs in the 70–79 year age group were smaller than those in the < 70 group. There were no significant differences between the 70–79 year age group and ≥ 80 years group.

**Table II:** Cardiac examinations by echocardiography and plasma test in the 3 age groups

Variables	< 70 years		70–79 years		80 years and above	
	Missing number	(*n* = 303)	Missing number	(*n* = 149)	Missing number	(*n* = 59)
LAD, mm	81	39.2 (33.3–44.2)	58	39.0 (36.2–42.9)	20	40.2 (37.5–44.2)
LVDd, mm	82	49.0 (44.5–55.6)	58	45.8 (41.2–51.0)*	19	48.7 (43.7–54.9)
LVDs, mm	82	34.3 (28.3–43.6)	58	30.7 (25.9–35.0)*	19	33.4 (28.1–39.6)
LVEF, %	81	59.2 (43.7–66.0)	58	63.8 (52.2–69.0)	19	57.8 (47.3–68.4)
SV, ml	131	55.0 (42.1–67.1)	79	53.5 (41.0–70.6)	27	55.0 (46.0–75.5)
TRPG, mmHg	146	21.2 (15.0–26.8)	73	22.4 (19.6–27.0)	23	23.0 (19.5–27.5)
BNP, pg/ml	109	52.1 (20.9–168.2)	58	103 (33–277)	19	111 (62–241)

Parameters obtained by echocardiography and the plasma level of BNP in the 3 age groups are shown.

* Indicates significant difference compared with the under 70 years group. LAD: left atrium diameter, LVDd: left ventricular end-diastolic diameter, LVDs: left ventricular end-systolic diameter, LVEF: left ventricular ejection fraction, SV: stroke volume, TRPG: tricuspid regurgitation peak gradient.

### Differences in the results of cardiopulmonary exercise test up to anaerobic threshold among the 3 age groups

**Fig. 1:**
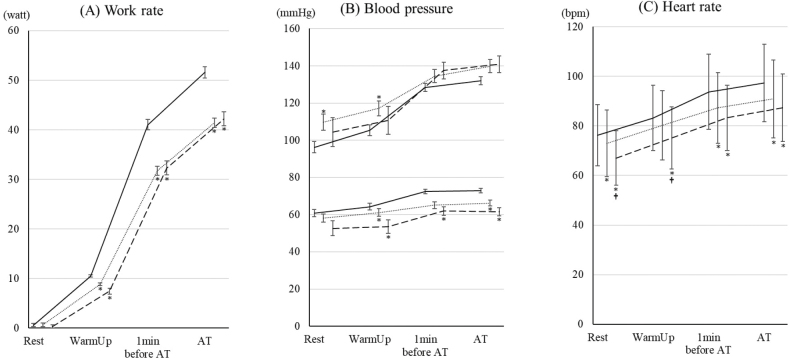
Changes in work rate and haemodynamic parameters during cardiopulmonary exercise test (CPET) up to aerobic threshold (AT) in the 3 age groups. The changes in (A) work rate, (B) blood pressure, and (C) heart rate during CPET up to AT in patients under 70, aged 70–79 years age, and ≥ 80 years are shown. The data are shown as the mean and standard error. Solid, dotted, and dashed lines show the under-70, 70–79 years, and ≥ 80 years groups, respectively. *Indicates a significant difference compared with the < 70 group. †Indicates a significant difference compared with the 70–79 year age group.

The changes in work rate and haemodynamics are shown in Fig. 1. The work rate values in the 70–79 year age group and ≥ 80 years group were lower than those in the < 70 group (Fig. 1A). Systolic blood pressure at rest and in the warmup periods in the 70–79 year age group was higher than those in the < 70 group (Fig. 1B). Diastolic blood pressure values in the 70–79 year age group and the ≥ 80 years group were lower than those in the < 70 group. There were no significant differences in blood pressure between the 70–79 year age group and ≥ 80 years group. Heart rate values in the 70–79 year age group and ≥ 80 years group were lower than those in the < 70 group. Heart rate values at rest and in the warmup periods in the ≥ 80 years group were lower than those in the 70–79 year age group.

**Fig. 2:**
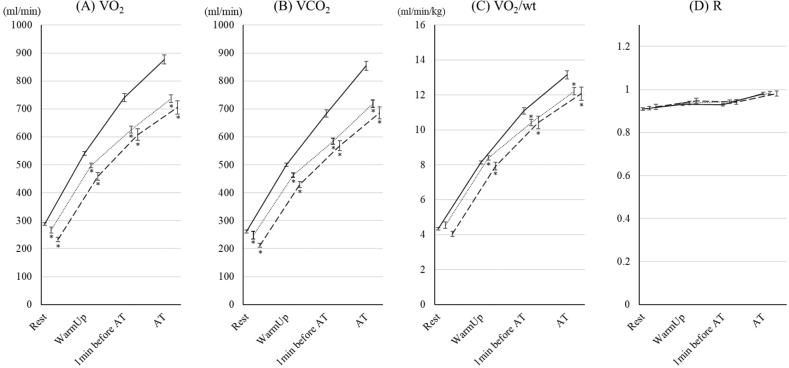
Changes in gas exchange parameters during cardiopulmonary exercise test (CPET) up to aerobic threshold (AT) in the 3 age groups. The changes in (A) oxygen consumption (VO_2_), (B) carbon dioxide output (VCO_2_), (C) VO_2_/wt; oxygen consumption per bodyweight (VO_2_/wt), and (D) respiratory exchange ratio ® during CPET up to AT in patients under 70, aged 70–79 years, and ≥ 80 years are shown. Data are shown as the mean and standard error. Solid, dotted, and dashed lines show the under-70, 70–79 years, and ≥ 80 years groups, respectively. *Indicates a significant difference compared with the < 70 group.

The changes in gas exchanges are shown in Fig. 2. There were no significant differences in these variables between the 70–79 year age group and the ≥ 80 years group. VO_2_ and VCO_2_ in the 70–79 year age group and the ≥ 80 years group were lower than those in the < 70 group. There were no significant differences in RER among the groups. VO_2_/wt values in the 70–79 year age group and ≥ 80 years group were lower than those in the < 70 group. There were no significant differences between the 70–79 year age group and the ≥ 80 years group.

**Fig. 3:**
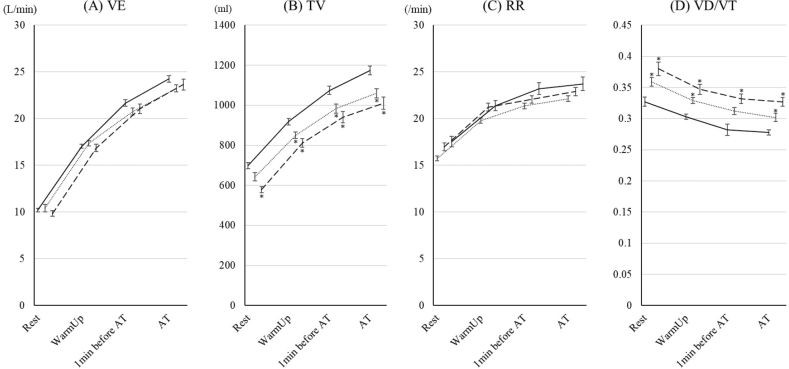
Changes in ventilatory volume parameters during cardiopulmonary exercise test (CPET) up to aerobic threshold (AT) in the 3 age groups. The changes in (A) ventilator equivalents (VE), (B) tidal volume (TV), (C) respiratory rate (RR), and (D) ratio of dead space to tidal volume (VD/VT) during CPET up to AT in patients under 70, aged 70–79 years, and ≥ 80 years are shown. The data are shown as the mean and standard error. Solid, dotted, and dashed lines show the under-70, 70–79 years, and ≥ 80 years groups, respectively. *Indicates a significant difference compared with the < 70 group.

**Fig. 4:**
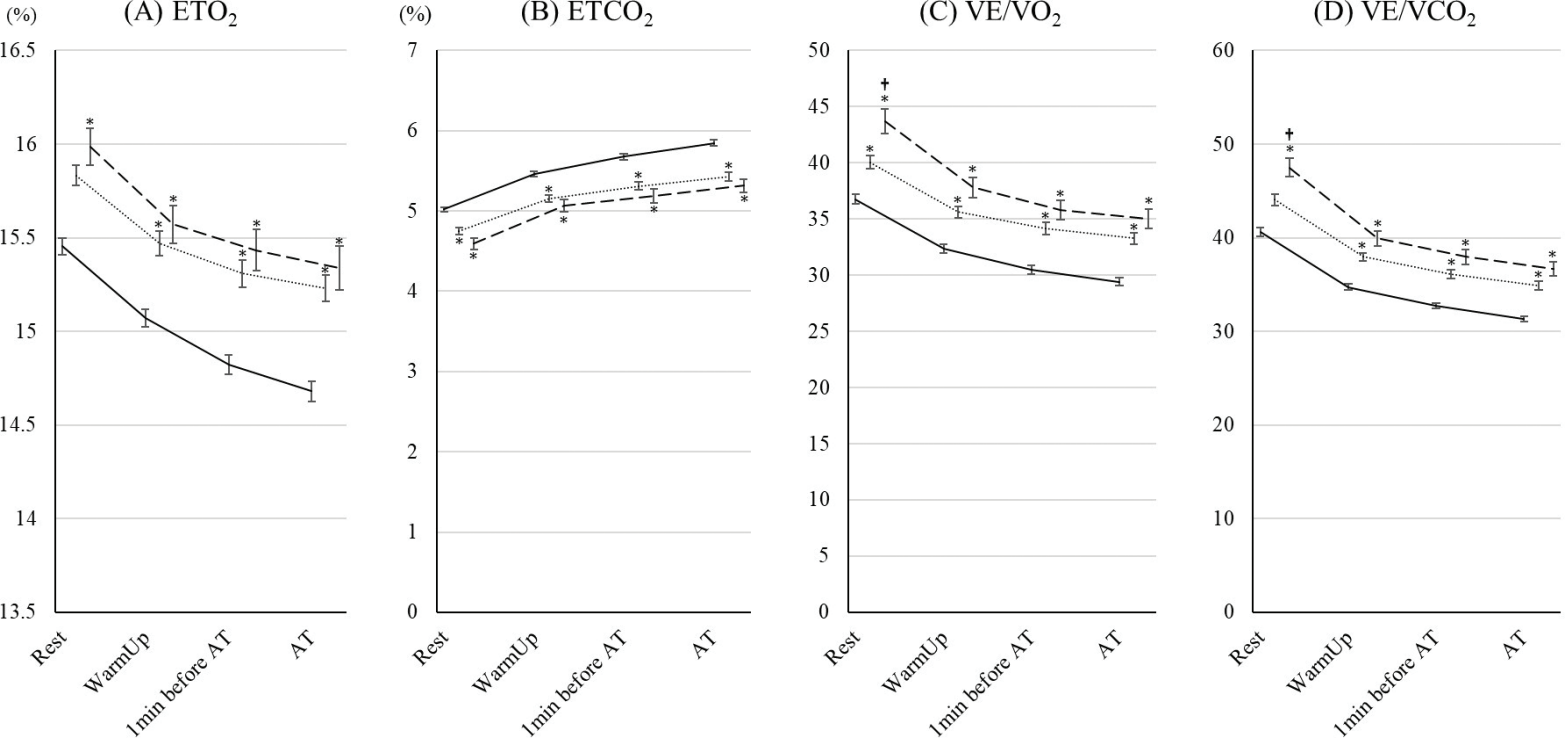
Changes in ventilatory efficiency parameters during cardiopulmonary exercise test (CPET) up to aerobic threshold (AT)in the 3 age groups. The changes in (A) end-tidal oxygen (ETO_2_), (B) end-tidal carbon dioxide (ETCO_2_), (C) ventilatory equivalents for oxygen (VE/VO_2_), and (D) ventilatory equivalents for carbon dioxide (VE/VCO_2_) during CPET up to AT in patients under 70, aged 70–79 years, and ≥ 80 years are shown. The data are shown as the mean and standard error. Solid, dotted, and dashed lines show the under-70, 70–79 years, and ≥ 80 years groups, respectively. *Indicates a significant difference compared with the < 70 group. †Indicates a significant difference compared with the 70–79 years group.

The changes in ventilatory volumes are shown in Fig. 3. There were no significant differences in these variables between the 70–79 year age group and the ≥ 80 years group. TV and VD/VT in the 70–79 year age group and the ≥ 80 years group were lower and higher than those in the < 70 group; however, there were no significant differences in VE between the groups. The changes in ventilatory efficiency are shown in Fig. 4. For all of the variables, ventilatory efficiency in the 70–79 year age group and the ≥ 80 years group was worse than for those in the < 70 group. In the ≥ 80 years group, VE/VO_2_ and VE/VCO_2_ at rest were significantly worse than for those in the 70–79 year age group.

## DISCUSSION

In this study, we evaluated the usefulness of CPET up to AT in older patients with CVD. Fifty-nine CVD patients aged ≥ 80 years performed CPET in our hospital over 9 years. In the ≥ 80 years group, the proportions of patients with chronic heart failure and ischaemic heart disease were high and the proportions of patients treated by percutaneous coronary intervention and cardiac operation were high and low, respectively. In CPET, most of the parameters in the 70–79 year age group and the ≥ 80 years group were significantly different than those in the < 70 group. However, in comparison with the 70–79 year age group and the ≥ 80 years group, only ventilator efficiency and heart rate decreased with age, and most of the other parameters including work rate and oxygen consumption per body weight were not significantly different between the groups. Even for CVD patients in the ≥ 80 years group, CPET up to AT can supply useful information for cardiac rehabilitation, as in the 70–79 year age group.

As the population continues to age, we should expect to observe that patients with CVD are also ageing. Thus, it is important not only to treat the disease but also to prevent frailty and sarcopenia and maintain the patient’s quality of life. Therefore, the demand for cardiac rehabilitation that can comprehensively respond to these needs is increasing, even among older patients (12–15). When we perform cardiac rehabilitation for older patients with CVD, it is very important to determine an appropriate exercise intensity with CPET in advance and prescribe tailored exercise. However, CPET has not been evaluated in people over the age of 80 years (16). A study of CPET in patients with heart disease, including those over age 80 years, showed that CPET can be used to safely evaluate athletic performance even in older patients with heart disease (17). It has been unclear whether it is effective for determining exercise intensity in patients over the age of 80.

Regarding baseline characteristics, there were many differences between the < 70 years group and the 70–79 year age group and ≥ 80 years group. The percentages of male, BMI, smoking, underlying disease, medication, and treatment in the < 70 years group were significantly different from those in the 70–79 year age group and the ≥ 80 years group. However, in comparison with the 70–79 year age group and the ≥ 80 years group, there were no significant differences in the use of beta blockers, LVEF, stroke volume, TRPG, or the plasma level of BNP. There were no major differences in the baseline characteristics in the 70–79 year age group or the ≥ 80 years group.

In this study, while there were many significant differences in the results of CPET up to AT between the < 70 years group and both the 70–79 year age group and ≥ 80 years group, almost none of the parameters were significantly different between the 70–79 year age group and the ≥ 80 years group. Work rate, VO_2_, VCO_2_, VO_2_/wt, and RER in the 70–79 year age group and the ≥ 80 years group changed in a similar way. CVD patients aged ≥ 80 years should be able to perform CPET up to AT, as in patients aged 70–79 years. There were no significant differences in the results regarding ventilatory volume during CPET up to AT between the 70–79 year age group and the ≥ 80 years group. Some parameters of ventilatory efficiency showed a poor outcome in the ≥ 80 years group compared with the 70–79 year age group. VE/VO_2_ and VE/VCO_2_ at rest in the ≥ 80 years group were significantly higher than those in the 70–79 year age group. A high VE/VO_2_ and VE/VCO_2_ indicate poor ventilatory efficiency. It has been reported that ageing worsens pulmonary diffusion capacity (18). This could have affected our study results. However, in the exercise periods, the differences in VE/VO_2_ and VE/VCO_2_ between the 70–79 year age group and the ≥ 80 years group decreased and were no longer significant. Even in patients aged ≥ 80 years the exercise intensity can be determined by CPET. The determination of an appropriate exercise intensity would be effective for promoting a healthy life.

Regarding haemodynamic parameters during CPET, heart rate values in the 70–79 year age group and the ≥ 80 years group were lower than those in the < 70 group. In addition, heart rate at rest and during warmup in the ≥ 80 years group were lower than those in the 70–79 year age group. As reported by Arbeev, the resting pulse rate in older healthy individuals decreases with ageing (19). These effects of ageing could have affected our study results. Diastolic blood pressure values in the 70–79 year age group and the ≥ 80 years group were lower than those in the < 70 group. Atherosclerosis has a bidirectional link with an increased pulse pressure and a decreased diastolic blood pressure (20). Diastolic blood pressure in older patients should decrease because atherosclerosis progresses with ageing (21). While some reports have shown that systolic blood pressure increases with age in healthy subjects (22), our results did not. Our results may have been affected by the fact that our study targeted CVD patients who had cardiac dysfunction and who took medication including beta blockers.

### Limitations

This study has several limitations. First, we did not evaluate the parameters over AT or the results of an interval analysis using peak period, such as VE/VCO_2_ slope, because many older patients showed a lower peak respiratory exchange ratio. Second, this was a retrospective study from a single centre. Additional multicentre studies in older patients will be needed. Third, we evaluated only older patients who performed CPET. The results of this study may not apply to all older patients, such as those with low physical strength. Fourth, we did not compare the results of CPET by sex due to the small sample size. However, we did find that there were no significant differences between the 70–79 year age group and the ≥ 80 years group. Fifth, we could not evaluate the daily physical activity of patients, such as leg muscle strength and power, which may have affected exercise capacity, because of a lack of data.

### Conclusion

CPET up to AT is useful for determining exercise intensity even in patients with CVD who are more than 80 years old.
